# CX3CL1/CX3CR1 signal mediates M1-type microglia and accelerates high-altitude-induced forgetting

**DOI:** 10.3389/fncel.2023.1189348

**Published:** 2023-05-10

**Authors:** Xueting Wang, Yuqi Xie, Yun Niu, Baolan Wan, Yapeng Lu, Qianqian Luo, Li Zhu

**Affiliations:** Institute of Special Environmental Medicine, Co-Innovation Center of Neuroregeneration, Nantong University, Nantong, China

**Keywords:** CX3CL1/CX3CR1 signal, M1-type microglia, high-altitude exposure, synaptic plasticity, cognition

## Abstract

**Introduction:**

Hypoxia-induced neuronal damage is the primary cause of cognitive impairment induced by high-altitude exposure. Microglia play a crucial regulatory role in the central nervous system (CNS) homeostasis and synaptic plasticity. M1-type polarized microglia are suspected to be responsible for CNS injury under hypoxic conditions, but the exact molecular mechanism is still unelucidated.

**Methods:**

CX3CR1 knock out and wide type mice were exposed to a simulated plateau at 7000 m for 48 h to construct the model of hypobaric hypoxia-induced memory impairment. The memory impairment of mice was assessed by Morris water maze. The dendritic spine density in the hippocampus was examined by Golgi staining. The synapses in the CA1 region and the number of neurons in the DG region were examined by immunofluorescence staining. The synapses in microglia activation and phagocytosis were examined by immunofluorescence. The levels of CX3CL1/CX3CR1 and their downstream proteins were detected. CX3CR1 knockout primary microglia were treated with CX3CL1 combined with 1% O_2_. The levels of proteins related to microglial polarization, the uptake of synaptosome and phagocytotic ability of microglia were detected.

**Results:**

In this study, mice exposed to a simulated 7000 m altitude for 48 h developed significant amnesia for recent memories, but no significant change in their anxiety levels was observed. Hypobaric hypoxia exposure (7000 m altitude above sea level for 48 h) resulted in synapse loss in the CA1 region of the hippocampus, but no significant changes occurred in the total number of neurons. Meanwhile, microglia activation, increased phagocytosis of synapses by microglia, and CX3CL1/CX3CR1 signal activation were observed under hypobaric hypoxic exposure. Further, we found that after hypobaric hypoxia exposure, CX3CR1-deficient mice showed less amnesia, less synaptic loss in the CA1 region, and less increase in M1 microglia, compared to their wildtype siblings. CX3CR1-deficient microglia did not exhibit M1-type polarization in response to either hypoxia or CX3CL1 induction. Both hypoxia and CX3CL1 induced the phagocytosis of synapses by microglia through the upregulation of microglial phagocytosis.

**Discussion:**

The current study demonstrates that CX3CL1/CX3CR1 signal mediates the M1-type polarization of microglia under high-altitude exposure and upregulates microglial phagocytosis, which increases the phagocytosis of synapses in the CA1 region of the hippocampus, causing synaptic loss and inducing forgetting.

## Introduction

With the increasing necessity of people to travel, work, and explore in highlands, approximately 140 million people currently live at altitudes above 2,500 m for extended periods. This triggers a range of high-altitude illnesses such as cerebral edema, pulmonary edema, acute altitude sickness, and cognitive impairment (Garrido et al., 2021). Furthermore, high-altitude exposure leads to delayed cognitive reaction time, decreased attention span, decreased executive ability, and loss of working memory (Koester-Hegmann et al., 2018). Cognitive impairment is positively correlated with the altitude level and the duration of exposure (Li and Wang, 2022). However, the mechanism of cognitive impairment caused by hypobaric hypoxia (HH) is still unclear. Researchers have suggested that cognitive impairment by high-altitude exposure is due to hypoxia which occurs when brain tissue consumes large amounts of oxygen. Exposure to hypoxia increases the release of excitatory and inhibitory synaptic transmitters in neurons, causing excitability, neuronal damage, and even brain cell death (Revah et al., 2016). HH exposure activates neuronal apoptotic signaling and triggers neuronal apoptosis (Shi et al., 2022). Neuronal damage directly affects the synthesis, uptake, and release of central neurotransmitters, which in turn affects interneural excitatory neurotransmitter transmission and synaptic plasticity, thus causing cognitive impairment (Sharma et al., 2019). A research has indicated that synaptic plasticity is one of the key processes that regulate learning and memory (Mayne and Burne, 2019). Changes in synaptic strength and behavior may arise from signaling in immune-related pathways, especially in those regulated by microglia (Wu et al., 2015).

Microglia constitute 5–12% of the cells of the central nervous system (CNS) (Hickman et al., 2018). They are the main resident immune cells of the brain and play a crucial role in regulating neuronal activity and synaptic plasticity (Elmore et al., 2018). Functional impairment of microglia is closely related to cognitive impairment. Acute brain trauma leads to impaired cognitive function, and this impairment is accompanied by microglia activation, which is positively correlated with impaired cognitive function (Saba et al., 2021). After the intraperitoneal injection of lipopolysaccharide, a significant increase in activated microglia was observed in the hippocampal region of mice with impaired cognitive function. In contrast, the administration of baicalin caused a reduction of activated microglia and inflammatory factors in the hippocampal region and an improvement in the cognitive function of mice, suggesting that over-activated microglia caused damage to the CNS (Li et al., 2020). After the removal of activated microglia, cognitive abilities were restored in mice in the Down’s syndrome model (Pinto et al., 2020). This indicated a protective effect of the inhibition of activated microglia on cognitive impairment in pathological conditions. Microglia activation is a major component of neuroinflammation in the CNS, providing the first line of defense in the event of injury or disease (Tang and Le, 2016). Under hypoxic conditions in the brain, Microglia polarized to the M1 type lead to the release of reactive oxygen species and pro-inflammatory factors, inducing neuroinflammation (Butturini et al., 2019). Hypoxia increased the release of iNOS and tumor necrosis factor α (TNFα) by microglia by stimulating the NF-κB pathway (Guo and Bhat, 2006; Liu L. R. et al., 2020). We have previously reported that HH exposure significantly activates microglia, increases the release of pro-inflammatory factors, and causes neurotoxicity. Moreover, HH exposure leads to increased phagocytosis of microglia (Wang X. et al., 2022).

Microglia directly sense synaptic activity (Cardona et al., 2006) and participate in synaptic pruning through phagocytosis (Sellgren et al., 2019). The depletion of microglia reduces learning-related synaptic structural plasticity and alters synaptic protein levels (Parkhurst et al., 2013). Resting microglia monitor the brain microenvironment by continuously extending and contracting branches, and reshaping neural circuits by forming synaptic communication with adjacent neurons (Zhao et al., 2017). In addition, microglia actively phagocytose and eliminate synaptic material during development and thus play a key role in synaptic pruning during postnatal development in mice. This suggests that defective microglia function may contribute to synaptic abnormalities in some neurodevelopmental disorders (Paolicelli et al., 2011). C-X3-C Motif Chemokine Receptor 1 (CX3CR1) is a G-protein-coupled receptor with a seven-times transmembrane structure and is uniquely expressed by microglia (Kim et al., 2011). Chemokine ligand 1 (CX3CL1), which is released by neurons, directly binds to CX3CR1 on microglia membranes and activates downstream signals related to microglia polarization (Zhang et al., 2018). CX3CL1/CX3CR1 are essential for the maintenance of homeostasis in the brain, and they significantly influence microglia activation by regulating inflammatory factors such as TNFα, Interleukin (IL)-6, IL-1β, and IL-10 to improve the inflammatory response in the brain (Laing et al., 2010). Adult CX3CR1 deficient mice showed increased autonomic neurotoxicity (Cardona et al., 2006; Reemst et al., 2016). It has been reported that microglia are involved in the phagocytosis of synapses through CX3CR1 or complement receptor 3 signal (Schafer et al., 2012). Basilico et al. (2019) reported reduced glutamate release and abnormal microglia function in CX3CR1 knockout mice. This demonstrates the importance of CX3CL1/CX3CR1 in microglia-neuron interactions for synaptic function and maturation (D’Haese et al., 2012; Basilico et al., 2019).

Based on the role of microglia in synaptic pruning and their hypoxic response characteristics, we hypothesized that HH exposure might trigger cognitive impairment by promoting microglia to M1-type, exacerbating neuroinflammation and synaptic loss. This study aimed to investigate the effects of HH exposure on the CX3CL1/CX3CR1 signal in microglia and to explore the regulatory mechanisms of this pathway in HH-induced forgetting.

## Materials and methods

### Animals and treatments

CX3CR1*^GFP/+^*mice [005582, B6.129P2 (Cg)-Cx3cr1^*TM*1Litt^/J, Jackson Laboratory] and wild-type control (C57BL/6) aged 6–8 weeks were used for this study. CX3CR1^GFP/GFP^ mice exhibiting CX3CR1 deficiency (KO) were bred from CX3CR1^GFP^

^/+^ mice. Animals were maintained at 23 ± 2°C with 45–60% humidity on a standard 12 h light/dark cycle. After being trained in the Morris Water Maze (MWM), the mice were divided into two groups: a normobaric normoxia (NN) group and a HH group. We constructed a HH animal model according to published studies (Lu et al., 2022; Wang X. et al., 2022) by exposing mice to a 60 cm × 40 cm × 40 cm HH chamber (ProOx-810, Shanghai Tawang Technology Co., Ltd.) and adjusting the air pressure inside the chamber to simulate different altitudes. The mice ascended from the sea level to 7000 m at a speed of 5 m/s and after 48 h, descended to the sea level at a speed of 5 m/s. The temperature and humidity inside the chamber were maintained at 20 ± 2°C and 50–60%, respectively. After HH exposure, the mice were euthanized and perfused with 0.9% saline via the left ventricle to remove the blood. The protocols of our study were reviewed and approved by the Animal Care and Use Committee of Nantong University (Approval ID: S20220219-007).

### MWM test

The MWM experiments were performed based on the previously reported methods (Sacks et al., 2018; Wu et al., 2022) with modifications required for the current experiments. Before HH exposure, the mice underwent a 7-day training trail. They were placed in a 150 cm-diameter circular pool with water temperature maintained at 22 ± 1°C. A 10-cm diameter circular escape platform was placed in the middle of the southwest quadrant and kept 1 cm below the water surface. The water was made with a non-toxic white opaque pigment. The mice had 90 s to find the platform and were allowed to stay on it for 30 s. On the 8th day, a probe trail was traced out as follows: The hidden platform was removed from the destination quadrant. The mice were released in the northeast quadrant and allowed free-swimming for 90 s. Then mice were exposed to HH and the probe trial was immediately repeated after 30 min of exposure. Any-Maze software was used to record and analyzed the swimming trajectory of each mouse. The time when the mice first found the escape platform and the average swimming speed were recorded.

### Open field test (OFT)

The OFT was performed to assess anxious behavior as per the protocol reported by Kraeuter et al. (2019). After HH exposure, the mice were placed in a 50 cm × 50 cm × 50 cm white polyvinyl chloride box one by one and allowed to move freely for 5 min. An overhead camera was used to record the movement of the test animal in the peripheral (15 cm from the wall) and central zones (25 cm × 25 cm). The mice’s activities were automatically recorded using Any-Maze software to record the distance traveled, average speed, time spent in the central area, and the number of entries in the box.

### Elevated plus maze (EPM) test

The mice were subjected to an EPM (Yoshizaki et al., 2020) immediately after the OFT to assess anxiety-like behavior. They were placed at the four-arm junction of the maze (35 cm × 5 cm) and 50 cm above the ground. The trajectory of the mice in the apparatus, retention time in the open arm, and several entries were automatically recorded for 5 min using the Any-Maze software.

### Golgi staining

The histological examination of dendritic spines was performed using a Golgi staining assay to assess the HH exposure effects on the hippocampal dendritic spines of the mice (Lan et al., 2022; Wu et al., 2022). The Golgi staining kit (Hito Golgi-Cox OptimStain™ PreKit, Hitobiotec Corp.) was used for the experiments. The test animals were anesthetized and their fresh, unperfused brains were promptly removed. After soaking the 1 cm-sized tissue in buffers as required by the protocol, the brains were cut into 100-μm coronal sections using a vibrating microtome (LEICA VT 1000S). Then, the resultant samples were stained and sealed. Images of slides were recorded using a Leica DM4000B microscope. The number of dendritic spines per 10 μm dendrite was quantified as the density of dendritic spines using FIJI ImageJ software (National Institutes of Health).

### Nissl staining

Nissler staining was applied for counting the mouse neurons (Wang J. et al., 2021) to assess the effect of HH exposure on the mice hippocampal neurons. Brain sections (40 μm) were soaked in trichloromethane and then stained using 1% tar violet. Subsequently, the resultant samples were subsequently dehydrated by ethanol gradient and xylene transparent. Then, they were imaged using a microscope Leica DM4000B.

### Primary microglia culture and treatments

As previously described (Wong et al., 2020; Chen et al., 2022; Wang X. et al., 2022), primary microglia were prepared from cerebral cortices of 2-day-old mice. After the removal of the meninges, cortical tissue was digested into single cells. Then cells were cultured in Dulbecco’s modified Eagle’s medium -F12 (10565018, Thermo Fisher Scientific) supplemented with 10% fetal bovine serum (10100147, Thermo Fisher Scientific), 5 ng/ml Granulocyte-macrophage colony-stimulating factor (78017, STEMCELL Technologies) and penicillin/streptomycin (100 U/ml and 100 mg/ml, respectively) at 37°C in a 5% CO_2_ humidified incubator. After 10 days, the mixed cells dominated by astrocytes formed a fused trophoblast. The microglia gradually increased and floated in the supernatant. Cells from the supernatant were then harvested after 14 days and seeded in 12-well culture plates. Iba1 (ab5076, Abcam) immunostaining was used to characterize microglia with microglial cell purity of over 98%.

In this study, a hypoxic environment was generated by adjusting the environment to 1% O_2_ and 5% CO_2_ using a hypoxic workstation (Ruskinn Technologies, UK), and primary microglia were cultured in the hypoxic workstation for 24 h (Lu et al., 2022). In this study, primary microglia were treated with 50 ng/ml CX3CL1 (HY-P72686, MCE) for 4 h to investigate the effect of CX3CL1 on hypoxia-induced microglia activation.

### Primary neuron isolation and co-culture with microglia

Primary neurons were taken from fetal mice at 13–15 days of gestational age (E15-16) as previously described (Wu et al., 2017). After the removal of the brain membranes, cortical tissue was digested into single-cell suspensions. Primary neurons were cultured in Dulbecco’s modified Eagle’s medium -F12 (10565018, Thermo Fisher Scientific) supplemented with 10% fetal bovine serum (10100147, Thermo Fisher Scientific) at 37°C with 5% CO_2_. After 2 h, cells were switched to Neurobasal complete medium (21103049, Thermo Fisher Scientific) containing B-27 supplement (A3582801, Thermo Fisher Scientific) and penicillin/streptomycin (100 U/ml and 100 mg/ml, respectively). A total of 10 μM cytarabine (HY-13605, MCE) was added to the culture system after 72 h culture and incubated cells for 48 h to inhibit the overgrowth of glial cells, after which the culture medium was replaced by a half amount of Neurobasal medium every 48 h.

Primary microglia were seeded at a density of 2.5 × 10^5^/mL in neurons that had been grown for 14 days and co-treated with 1% O_2_ for 24 h to construct a hypoxia-treated co-culture cell system.

### Brain tissue synaptosome extraction

The published method for synaptosome extraction from brain tissue was modified as per our experimental needs (Carlin et al., 1980; Biesemann et al., 2014; Kallergi et al., 2022). A total of 1 g fresh brain tissue was well homogenized in a 10-fold buffer, and the supernatant was centrifuged at 800 × *g* for 10 min and aspirated. The supernatant was further centrifuged at 17,000 × *g* for 20 min and the precipitate was resuspended in 1.2, 1.0, and 0.8 mol/l sucrose gradient buffers. After centrifugation at 82,500 × *g* for 2 h, the liquid at the 1.0–1.2 mol/l sucrose interface was collected and the synaptosomes were diluted with 10 mM HEPES buffer (15630106, Thermo Fisher Scientific). Then synaptosome suspension was centrifuged at 15,000 × *g* for 30 min and the precipitate was resuspended in 4 ml Dulbecco’s modified Eagle’s medium -F12 (10565018, Thermo Fisher Scientific) medium, which was directly incubated with microglia within 30 min.

### Microglia phagocytosis assay

We used the amount of microglia-phagocytosing synaptosomes and TRITC-Dextran 40 kDa (AS026, Abclonal) (Xue et al., 2022) to assess the changes in the phagocytosis of microglia post CX3CL1 and hypoxia treatment. The synaptosome mixture was centrifuged at 150,000 × *g* for 30 min and the precipitate was resuspended in cell medium. Thereafter, primary microglia were separately incubated with 100 μg/mL TRITC-Dextran 40 kDa or synaptosome mixture for 30 min. The cells were then fixed and stained with a synaptosomal marker Synaptophysin antibody (101011, Synaptic Systems) using immunofluorescence. Images were recorded by a Leica SP8 confocal microscope to quantify the fluorescence intensity of synaptosomes or TRITC-Dextran in the cells.

### Protein isolation and western blotting

The samples were lysed by RIPA (P0013B, Beyotime, China) to extract proteins, the concentration of which was measured by bicinchoninic acid assay. Thereafter, the proteins were isolated and transferred to polyvinylidene fluoride membranes. Thereafter, these membranes were incubated with primary antibodies including anti-CD206 (24595T, CST), anti-CD16 (ab246222, Abcam), anti-IL-1β (ab9722, Abcam), and anti-β-actin (A5316, Sigma). The binding of primary antibodies was visualized using HRP-conjugated secondary antibodies and the ECL Plus system. Grayscale analysis was performed using FIJI ImageJ software (National Institutes of Health).

### Total RNA isolation and qRT-PCR

Total RNA was isolated by TRIzol reagent (15596026, Thermo Fisher Scientific). A total of 1 μg purified total RNA was reverse-transcribed by HiScript III 1st Strand cDNA Synthesis Kit (R323-01, Vazyme, China) following the manufacturer’s instructions. Thereafter, qRT-PCR was performed using AceQ qPCR SYBR Green Master Mix (Q141-02, Vazyme, China).

All primers used for qRT-PCR were designed as follows: *Il1b* forward: 5′-TGCCACCTTTTGACAGTGATG-3′, reverse: 5′-TGATGTGCTGCTGCGAGATT-3′; Il6 forward: 5′-CCACTT CACAAGTCGGAGGCTTA-3′ reverse: 5′-CCAGTTTGGTAGCA TCCATCATTTC-3′; *Tnfa* forward: 5′-AAGCCTGTAGCCCACGT CGTA-3′, reverse: 5′-GGCACCACTAGTTGGTTGTCTTTG-3′; *Tgfb* forward: 5′-CACAAGAGCAGTGAGCGCTGAA-3′; *Cx3cr1* forward: 5′-CAGTGGCTTTGCTCATCCGCTA-3′, reverse: 5′-AG CCTGGTGATCCAGATGCTTC-3′; *Cx3cl1* forward: 5′-CAGTGG CTTTGCTCATCCGCTA-3′, reverse: 5′-AGCCTGGTGATCCAG ATGCTTC-3′; *Actb* forward: 5′-CATCCGTAAAGACCTCTA TGCCAAC-3′, reverse: 5′-ATGGAGCCACCGATCCACA-3′. The relative amount of gene expression was calculated using ^△△^ Ct, where Ct denotes the PCR threshold cycle.

### Immunofluorescence staining and assay

Tissue sections (40 μm) and cultured cells were fixed with 4% paraformaldehyde. The resultant samples were subsequently permeabilized with 0.3% Triton x-100. After being blocked by 10% donkey serum, the brain sections and cells were separately incubated with the following antibodies: anti-postsynaptic density-95 (PSD95) (3409T, CST), anti-Synaptophysin (101011, Synaptic Systems), anti-Iba1 (ab5076, Abcam), and anti-lysosomal associated membrane protein 1 (LAMP1) (sc-19992, SANTA), anti-NeuN (MAB377, Merck). The primary antibody binding was visualized with Alexa Fluor 488-conjugated donkey anti-goat IgG (ab150129, Abcam), Alexa Fluor 488-conjugated donkey anti-rat IgG (712-545-150, Jackson), Alexa Fluor 555-conjugated donkey anti-rabbit IgG (ab150074, Abcam), Alexa Fluor 647-conjugated donkey anti-mouse IgG (ab150107, Abcam), and Alexa Fluor 488-conjugated donkey anti-mouse IgG (ab150107, Abcam), respectively. Thereafter, these samples were counterstained with 1 μg/ml DAPI (D9542, Sigma). Images were recorded using a Leica SP8 confocal microscope.

We analyzed the microglial circularity and microglia branching index using the ImageJ Shape descriptors tool and ImageJ Sholl analysis tool, respectively, following existing methods (Young and Morrison, 2018; Obadia et al., 2022). The branching index was quantified as follows: the microglia cytosol was centered and the analysis circle was drawn with the longest microglial radius as the endpoint. The soma areas and morphological index (MI) were quantified followed with existing methods (Tremblay et al., 2012). The number of intersections between microglia and the analysis circle was quantified by the Sholl plugin and that number represented the microglial branching index. Further, the co-localization analysis of PSD95 and LAMP1 was performed using the ImageJ Colocalization plugin in Coloc 2 Analysis.

### Statistical analysis

GraphPad Prism v8.0 (GraphPad) was used for data analysis, which included a Student’s *t*-test and one-way or two-way ANOVA followed by Dunnett’s multiple comparisons. The data were presented as mean ± S.D. The significance levels for all the graphs were as follows: *p < 0.05, ^**^p < 0.01, and ^***^p < 0.001, and n.s. indicates no significance.

## Results

### HH-induced forgetting and synaptic loss in the hippocampus are associated with the enhanced microglial phagocytosis of synapses

**FIGURE 1:**
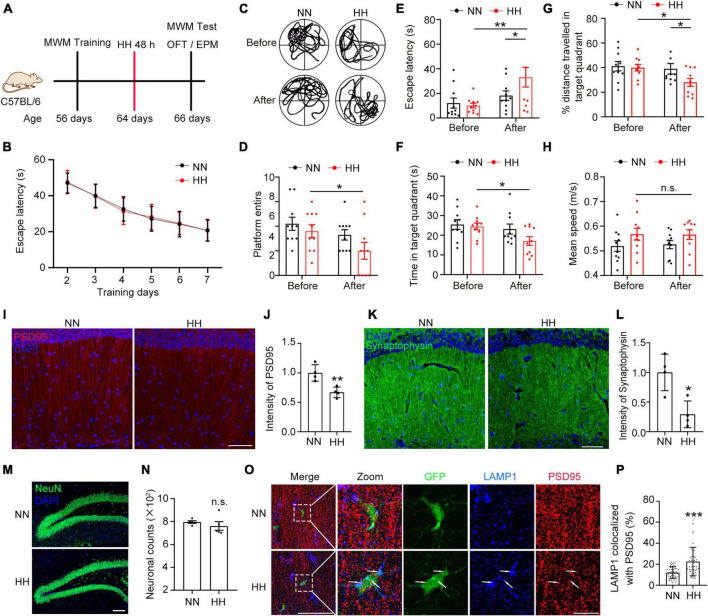
Hypobaric hypoxia (HH) exposure induces synaptic loss in hippocampus and amnesia. **(A)** C57BL/6 mice were trained by MWM and exposed to simulated altitude of 7000 m for 48 h, and their memory changes were assessed by MWM and their anxiety was detected by OFT and EPM. *n* = 10. **(B)** Escape latency of two groups of mice before HH exposure during the training trails. **(C)** Trajectories of the mice in the MWM probe trails before and after HH exposure. **(D)** Number of times mice entered into the platform in probe trails. **(E)** Escape latency of mice reaching the target platform. **(F)** Residence time of mice in the target quadrant. **(G)** Ratio of distance traveled by mice in the target quadrant. **(H)** Mean swimming speed of mice during the probe trails. **(I,J)** Immunofluorescence labeling of the postsynaptic membrane marker PSD95 in excitatory neurons in the CA1 region of the hippocampus (interaural 1.62 mm), Scale bar = 50 μm, *n* = 4. **(K,L)** Immunofluorescent labeling of Synaptophysin, a presynaptic membrane marker of neurons in the CA1 region of the hippocampus (interaural 1.62 mm), Scale bar = 50 μm, *n* = 4. **(M,N)** Immunofluorescence labeling of NeuN in hippocampal DG area neurons and cell counting was performed by Image J software, Scale bar = 100 μm, *n* = 5. **(O,P)** Immunofluorescence labeling of PSD95 and the lysosomal marker LAMP1 in hippocampal CA1 region (interaural 1.74 mm) in CX3CR1-GFP mice after exposure to HH for 48 h, Scale bar = 10 μm (*n* = 43 in NN group, *n* = 48 in HH group. Brain sections from 6 mice of each group were stained, and 5–8 cells of each mouse were quantified). *p < 0.05, **p < 0.01, ***p < 0.001, and n.s. indicates no statistical differences.

To investigate the effects of HH on cognitive function, 7-week-old mice (10 mice per group) were trained in a MWM for 7 days and then exposed to a simulated altitude of 7000 m for 48 h (Figure 1A). As shown in Figure 1B, the training curves of both groups of animals showed no difference. After HH exposure, mice were immediately subjected to the probe trail of MWM to study memory changes. Furthermore, OFT and EPM experiments were performed to assess anxiety alterations (Supplementary Figures 1A–H). The findings indicated that the swimming trajectory of mice was significantly disorganized after HH exposure (Figure 1C). In addition, the entries onto the platform were significantly reduced (Figure 1D) and the escape latency to reach the platform was significantly longer (Figure 1E) in post-exposure mice. Moreover, the residence time and swimming distance in the target quadrant were reduced after the HH exposure (Figures 1F, G). Conversely, average swimming speed was similar before and after exposure (Figure 1H). These results suggest that HH exposure induced the forgetting of recent memory in mice. However, in OFT, the dwell time and entries in the central region did not significantly change post-HH exposure (Supplementary Figures 1A–D). The EPM experiments indicated that the entries, moving distance, and dwell time in the open arms were unaltered after HH exposure (Supplementary Figures 1E–H). These findings suggested that HH exposure did not affect anxiety in mice.

Since CA1 neurons of the hippocampus are associated with the learning and memory functions in mice (Bartsch et al., 2011; Okuyama et al., 2016; Liu et al., 2017; Wang Y. et al., 2021), we further studied the neuronal changes in the CA1 region. The results demonstrated that the excitatory neuronal postsynaptic marker PSD95 was significantly reduced after HH exposure (Figures 1I, J). In addition, the presynaptic marker Synaptophysin was notably decreased (Figures 1K, L), suggesting that HH exposure caused loss of neuronal synapses. However, HH exposure did not change the number of neurons in the dentate gyrus (DG) region of the hippocampus (Figures 1M, N and Supplementary Figure 2), implying that the HH-induced reduction in synapses did not arise from a reduction in the number of neurons. Further, we observed that in the CA1 region of CX3CR1-GFP mice brain, the co-localization rate of LAMP1 with PSD95 was considerably increased in GFP^+^ cells post HH exposure (Figures 1O, P), which implies that HH-induced synaptic loss in the hippocampus is related to enhanced synaptic pruning by microglia.

### HH promotes microglia polarization to M1 type and release of pro-inflammatory factors through upregulation of CX3CL1/CX3CR1 signal

**FIGURE 2:**
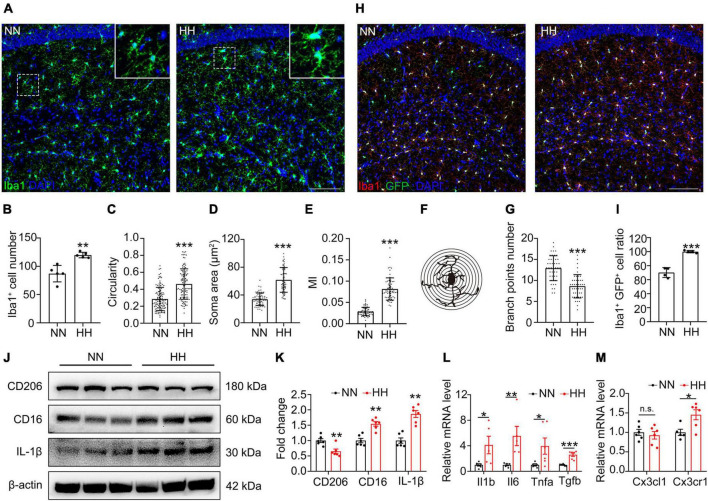
Hypobaric hypoxia (HH) exposure induces CX3CL1/CX3CR1 signal activation and M1-type polarization of hippocampal microglia. **(A)** Immunofluorescence labeling of microglia/macrophage marker Iba1 in CA1, Scale bar = 100 μm. **(B–G)** Statistics of panel **(A)**, including the number of Iba1^+^ cells [**(B)**, *n* = 5], circularity [**(C)**, *n* = 118, brain sections from 5 mice of each group were stained, and 23–24 cells of each mouse were quantified], soma area [**(D)**, *n* = 61 or 68, brain sections from 5 mice of each group were stained, and 12–13 cells of each mouse were quantified], MI [**(E)**, MI = soma area/arborization area, *n* = 62 or 64, brain sections from 5 mice of each group were stained, and 13–14 cells in CA1 region of each mouse were quantified] and number of branches [**(F,G)**, *n* = 45, brain sections from 5 mice of each group were stained, and 9 cells of each mouse were quantified]. **(H)** Immunofluorescence labeling of microglia/macrophage marker Iba1 in CA1 region of CX3CR1-GFP mice, Scale bar = 100 μm. **(I)** Statistics of the ratio of Iba1^+^GFP^+^ cells in GFP + cells in panel **(H)**, *n* = 4. **(J,K)** Western blot detection of CD206, CD16, and IL-1β levels in mouse hippocampus, *n* = 6. **(L)** qRT-PCR detection of the expression of inflammatory factors *Il1b*, *Il6*, *Tnfa*, and *Tgfb* in hippocampal tissue, *n* = 5. **(M)** qRT-PCR detection of *Cx3cl1* and *Cxcr1* expressions in hippocampal tissues, *n* = 6. *p < 0.05, **p < 0.01, ***p < 0.001, and n.s. indicates no statistical difference.

To explore the effects of HH exposure on microglia, the macrophage/microglia marker Iba1 in the CA1 region (Figure 2A) was labeled. The results revealed that HH significantly increased the number of Iba1^+^ cells, circularities, soma areas and MI (Figures 2B–E), and decreased the number of microglial branches (Figures 2F, G). These results indicated that HH significantly induced the proliferation and activation of macrophage/microglia. To confirm the source of the increased number of cells, we labeled Iba1 in the brains of CX3CR1-GPF mice after HH exposure. Consequently, the ratio of CX3CR1^+^Iba1^+^ cells was not decreased after HH exposure (Figures 2H, I), demonstrating that the rise in the Iba1^+^ cell population after HH exposure was due to the proliferation of microglia rather than the recruitment of peripheral macrophages. Further, we investigated the microglia polarization after HH exposure. As shown in Figures 2J, K, the M1-type microglia marker CD16 is significantly upregulated, while the M2-type marker CD206 was notably decreased in the hippocampal tissue after HH exposure. The proinflammatory factor IL-1β is also considerably increased, suggesting that HH caused M1-type microglial polarization. The expression of pro-inflammatory factors *Il1b*, *Tnfa*, and *Il6* was consistently upregulated, while the expression of anti-inflammatory factor *Tgfb* was significantly decreased (Figure 2L), demonstrating that microglia release pro-inflammatory factors after HH exposure and induce neuroinflammation. We further observed that the pro-inflammatory regulator CX3CR1 expression was substantially upregulated, while CX3CL1 stayed unaltered (Figure 2M), suggesting that the activation of the CX3CL1/CX3CR1 signal by HH-induced M1-type microglia. Furthermore, based on the CX3CR1-mediated “find me” signal (Bazan et al., 1997; Sokolowski et al., 2014; Szepesi et al., 2018; Puntambekar et al., 2022), we speculate that the activation of the CX3CL1/CX3CR1 signal is also involved in HH-induced microglial synaptic pruning.

### CX3CR1 deficiency attenuates HH-induced forgetting and synaptic loss

**FIGURE 3:**
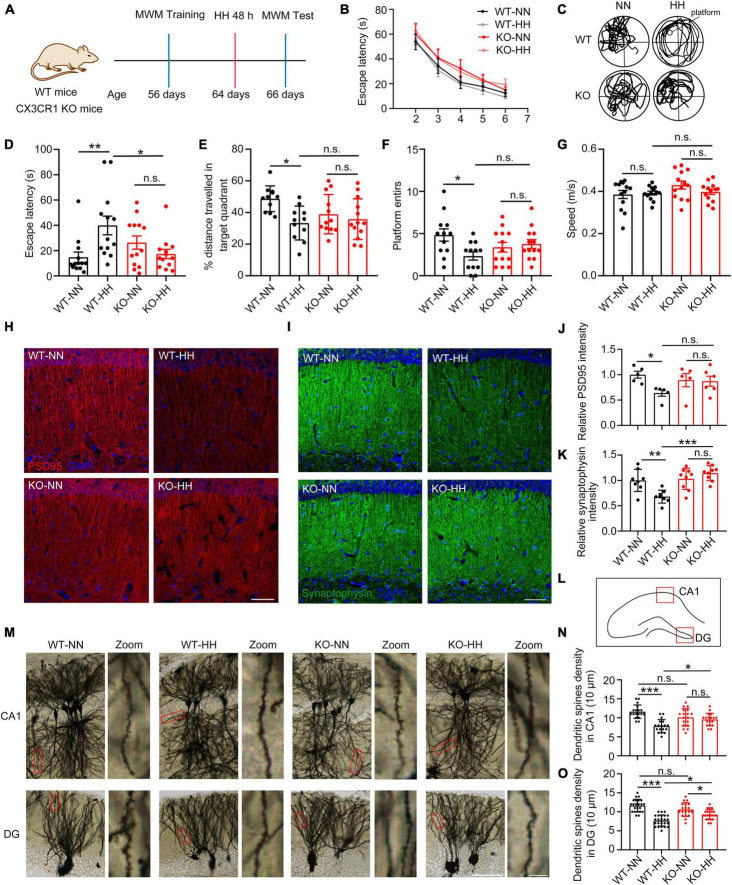
CX3CR1 deficiency attenuates HH exposure-induced synapse loss and amnesia. **(A)** Cx3CR1-deficient mice (KO) were trained with same-aged wide type (WT) mice by MWM, and then exposed to a simulated altitude of 7000 m for 48 h. The memory alterations were detected by MWM probe trail, *n* = 13. **(B)** Escape latency of four groups of mice before HH exposure during the training trails. **(C)** Trajectories of the mice in the MWM probe trails after HH exposure. **(D)** Escape latency of mice reaching the target platform after HH exposure. **(E)** The distance traveled by the mice in the target quadrant. **(F)** A number of times mice entered the platform. **(G)** Mean swimming speed of mice during the probe trails. **(H,J)** Immunofluorescence labeling of PSD95 in the CA1 region of the hippocampus (interaural 1.62 mm), Scale bar = 50 μm, *n* = 5. **(I,K)** Immunofluorescence labeling of Synaptophysin in the CA1 region of the hippocampus (interaural 1.62 mm), Scale bar = 5 μm, *n* = 8. **(L)** Image regions of panel **(M)** in mouse hippocampus. **(M)** Golgi staining labeling of neuronal spines in the hippocampal CA1 and DG regions of the mouse brain (interaural 1.74 mm). **(N,O)** Statistics of dendritic spine density in CA1 **(M)** and DG **(N)**, Scale bar = 25 μm, *n* = 18 (Brain sections from three mice of each group were stained, and six dendrites of each mouse in indicated region were quantified). *p < 0.05, **p < 0.01, ***p < 0.001, and n.s. indicates no statistical difference.

To study the influence of the CX3CL1/CX3CR1 signal in the effects of HH on cognitive function, CX3CR1-deficient (KO) mice (13 mice per group) were exposed to HH for 48 h after training in the MWM for 7 days. As shown in Figures 3A, B, the training curves of the animals were the same, and although the KO mice show some decrease in learning ability, the statistical differences are not significant. This may be owing to the young age of the mice that the CX3CR1 deficiency has not yet exerted a significant effect on cognitive function. Our results also reveal that WT mice demonstrated significantly non-directional motor trajectories post-HH exposure, whereas, in KO mice, the HH exposure had no notable effect on their motor trajectories (Figure 3C). The KO mice consistently, showed no noticeable changes in the escape latency to first reach the platform (Figure 3D), moving distance in the target quadrant (Figure 3E), entries onto the platform (Figure 3F), and swimming speed (Figure 3G) after HH exposure. These results imply that the deficiency of CX3CR1 effectively mitigates or even avoids HH-induced forgetting.

Further, we studied the post-HH exposure neural changes in the CA1 region of KO mice. The results indicated no notable HH exposure effect on the PSD95 and Synaptophysin levels of KO mice (Figures 3H–K). The lack of CX3CR1 effectively attenuated or even escaped the effect of HH on the synapses Consistent with the results in Figure 1, dendritic spine densities in CA1 and DG regions were significantly reduced in WT mice after HH exposure, whereas no noticeable changes were observed in KO mice (Figures 3L–O). These results suggest that in CX3CR1-deficient mice, HH lost its effect on the synapses in the CA1 region of the hippocampus, and thus, could not significantly influence the memory function.

### CX3CR1 deficiency reduces pro-inflammatory factors and phagocytosis of synapses by attenuating HH-induced M1-type polarization of microglia

**FIGURE 4:**
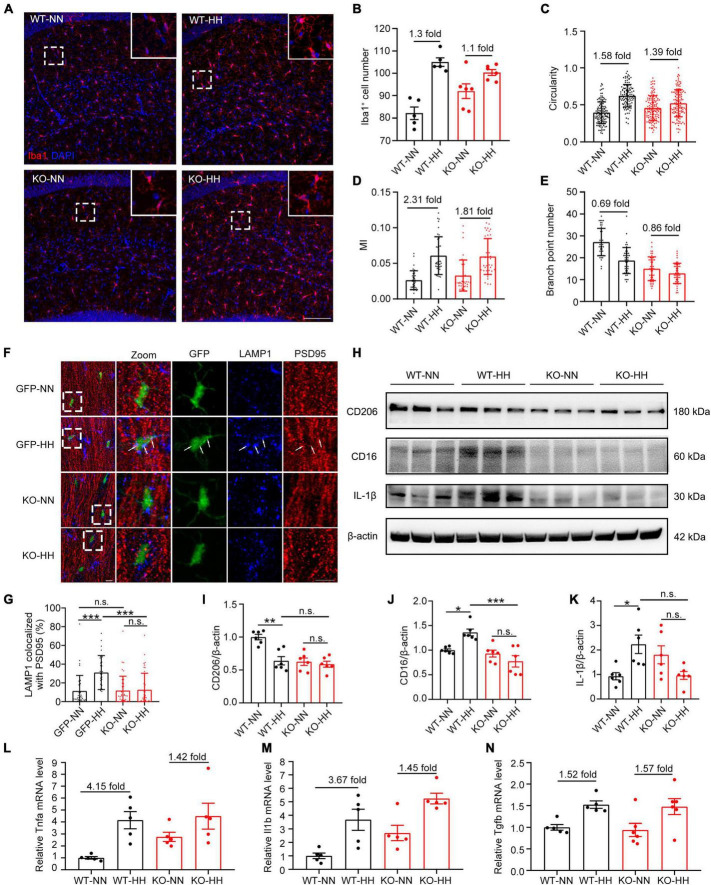
CX3CR1 deficiency attenuates HH-induced-M1-type microglia and phagocytosis of synapses by microglia. **(A)** Immunofluorescence labeling of microglia/macrophage marker Iba1 in the hippocampal CA1 region, Scale bar = 100 μm. **(B–E)** Statistics of panel **(A)**, including the number of Iba1^+^ cells [**(B)**, *n* = 5], circularity [**(C)**, *n* = 111–130, brain sections from 5 mice of each group were stained, and 20–30 cells of each mouse were quantified], MI [**(D)**, *n* = 33 to 43, brain sections from 5 mice of each group were stained, and 6 to 7 cells from CA1 regions of each mouse were quantified], and branch numbers [**(E)**, *n* = 45, brain sections from 5 mice of each group were stained, and 9 cells of each mouse were quantified]. **(F)** Immunofluorescence labeling of LAMP1 and PSD95 in the hippocampal CA1 region of CX3CR1-GFP and KO mouse, Scale bar = 10 μm. **(G)** Co-localization ratio of LAMP1 to PSD95 in GFP^+^ cells in panel **(F)** were determined. *n* = 35–41, brain sections from 6 mice of each group were stained, and 5–7 cells of each mouse were quantified. **(H–K)** Western blot detection of CD206, CD16, and IL-1β levels in mouse hippocampal tissue, *n* = 6. qRT-PCR was performed to detect the changes in the levels of inflammatory factors *Tnfa* **(L)**, *Il1b* **(M)**, and *Tgfb* **(N)** in the hippocampus, *n* = 5. *p < 0.05, **p < 0.01, ***p < 0.001, and n.s. indicates no statistical difference.

To investigate whether CX3CR1 deficiency attenuated HH-activated microglia, we stained Iba1 in HH-exposed KO or WT mouse brain sections (Figure 4A). The results showed that compared with WT group, the great increase of Iba1^+^ microglia, circularity and MI of microglia caused by HH were significantly reduced in CX3CR1 deficiency mice (Figures 4B–D). And correspondingly, the decrease of branching index was also significantly reduced (Figure 4E). These findings implied that CX3CR1 deficiency attenuates the activation of microglia by HH. Furthermore, the number of synapses engulfed by microglia was found to be reduced post-HH exposure in KO mice (Figures 4F, G), suggesting that the regulation of the engulfment of synapses by the microglia after HH exposure is dependent on CX3CR1. By investigating the changes of CD206, CD16, and IL-1β in the hippocampus of KO mice, it was found that the expression of microglia polarization markers remained unaffected by HH exposure (Figures 4H–K). The magnitude of changes in the pro-inflammatory factors *Tnfa* and *Il1b* were attenuated (Figures 4L, M), while the anti-inflammatory factor *Tgfb* was slightly upregulated (Figure 4N). These results suggest that HH-induced microglia polarization is dependent on the CX3CR1 signal, and CX3CR1 deficiency leads to an escape from HH-induced M1-type polarization and the secretion of pro-inflammatory factors in microglia. CX3CR1 may be involved in the switching of phenotype from M1 to M0.

### CX3CL1/CX3CR1 signal upregulates hypoxia-induced M1-type polarization of primary microglia

**FIGURE 5:**
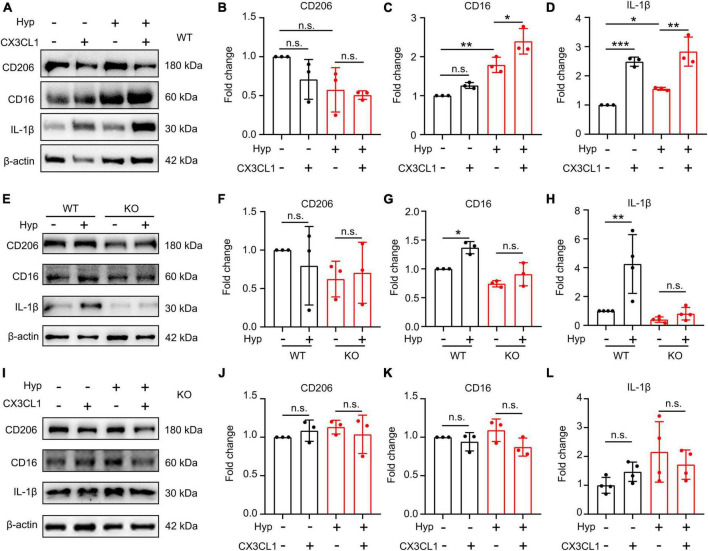
CX3CL1/CX3CR1 signal mediates hypoxia-induced-M1 type microglia. Primary microglia were treated with 1% O_2_ for 20 h followed by the co-treatment with 50 ng/ml CX3CL1 for 4 h. **(A–D)** Western blot detection of CD16, CD206, and IL-1β levels in microglia after hypoxia and CX3CL1 treatment **(A)**, and the grayscale values of each band were counted **(B–D)**, *n* = 3. **(E–H)** Primary microglia generated from wild-type (WT) or CX3CR1-deficient (KO) mice were treated with 1% O_2_ for 24 h. The cells were lysed to determine CD16, CD206, and IL-1β levels by Western blot, *n* = 3. **(I–L)** Primary KO microglia were treated with 1% O_2_ for 20 h followed by the co-treatment of 50 ng/ml CX3CL1 for 4 h. Cells were lysed to detect CD16, CD206, and IL-1β levels by Western blot, *n* = 3. *p < 0.05, **p < 0.01, ***p < 0.001, and n.s. indicates no statistical difference.

With an aim to investigate whether CX3CL1/CX3CR1 directly regulates hypoxia-induced microglia polarization, primary microglia were treated with 1% O_2_ for 20 h, and then were co-treated with CX3CL1 for 4 h. As shown in Figures 5A–D, hypoxia had no significant effect on CD206 in primary microglia, but upregulated CD16 and IL-1β, suggesting that hypoxia treatment induces the M1-type polarization of microglia. After CX3CL1 administration, microglia polarization marker expression was not significantly altered, but IL-1β was notably increased, suggesting that the activation of CX3CL1/CX3CR1 signal-induced M1-type microglia. The further upregulation of CD16 and IL-1β was observed after combined treatment of both hypoxia and CX3CL1, demonstrating that CX3CL1/CX3CR1 signal could exacerbate hypoxia-induced M1-type microglia and release pro-inflammatory factors.

To confirm whether the effect of hypoxia on microglia polarization is dependent on the CX3CR1 signal, we subjected KO microglia to combined hypoxia and CX3CL1 treatment. As shown in Figures 5E–H, hypoxia treatment did not affect polarization and synthesis of the pro-inflammatory factor IL-1β in KO microglia. The addition of CX3CL1 also fails to alter microglial polarization and IL-1β secretion under hypoxic conditions (Figures 5I–L). These findings demonstrate the dependence of hypoxia-induced M1-type polarization of primary microglia on the activation of the CX3CL1/CX3CR1 signal.

### CX3CL1/CX3CR1 signal upregulates hypoxia-induced microglial phagocytosis of synapses by promoting microglial endocytosis

**FIGURE 6:**
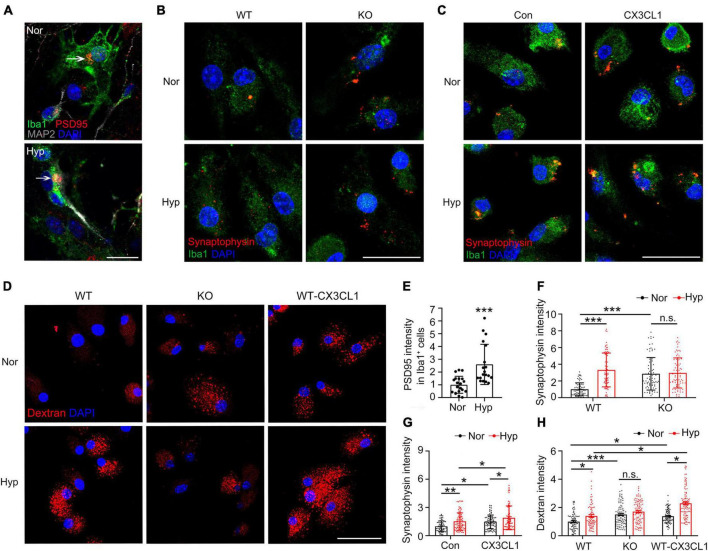
CX3CL1/CX3CR1 signal mediates hypoxia-induced phagocytosis of synapses by microglia. **(A)** Primary neurons co-cultured with microglia were treated with 1% O_2_ for 24 h. Iba1 and MAP2 with PSD95 were labeled by Immunofluorescence. **(B)** CX3CR1-deficient (KO) microglia were treated with 1% O_2_ for 24 h and incubated with dissociated synapses for 30 min. Immunofluorescence labeling of Iba1 and Synaptophysin. **(C)** Primary microglia were treated with 1% O_2_ for 20 h followed by the co-treatment of CX3CL1 for 4 h and incubated with dissociated synapses for 30 min. Immunofluorescence labeling of Iba1 and Synaptophysin. **(D)** WT and KO microglia were treated with 1% O_2_ for 24 h. WT microglia were treated with 1% O_2_ for 20 h followed by CX3CL1 co-treatment for 4 h. Then, the cells were incubated with TRITC-Dextran 40 kDa for 30 min, and confocal images were recorded. **(E)** Statistics of the total fluorescence intensity of PSD95 in Iba1^+^ microglia in panel **(A)**, *n* = 18. **(F)** Statistics of the total fluorescence intensity of Synaptophysin in Iba1^+^ microglia in panel **(B)**, *n* = 80. **(G)** Statistics of the total fluorescence intensity of Synaptophysin in Iba1^+^ microglia in panel **(C)**, *n* = 55–78. **(H)** Statistics of the total fluorescence intensity of TRITC-Dextran in single cells in panel **(D)**, *n* = 85–100. Scale bar = 20 μm, *p < 0.05, **p < 0.01, ***p < 0.001, and n.s. indicates no statistical difference.

We constructed a primary microglia-neuron co-culture system to explore the direct regulatory role of hypoxia in microglial phagocytosis of synapses. As shown in Figures 6A, E, the PSD95 signal in microglia increased significantly post-hypoxia, suggesting that hypoxia promoted the phagocytosis of synapses by microglia. Further, we incubated synaptosomes with KO microglia after hypoxia treatment. As shown in Figures 6B, F, the ability of KO microglia to phagocytose synaptosomes was significantly increased compared to WT microglia, but there was no noticeable change post-hypoxia treatment. Furthermore, the results presented in Figures 6C, G also showed that either hypoxia or CX3CL1 treatment significantly improved the microglial ability to phagocytose synapses, and the combined treatment further improved this ability. The aforementioned results demonstrate that the effect of hypoxia on microglial phagocytosis of synapses is CX3CL1/CX3CR1 signal-dependent.

To confirm whether the hypoxia-upregulated phagocytosis of synapses by microglia is related to endocytosis, WT and KO microglia were co-treated with hypoxia combined with CX3CL1 after incubation with the substrate TRITC-Dextran 40 kDa. As shown in Figures 6D, H, hypoxia treatment upregulated the endocytosis of WT microglia, while such an effect is absent in KO microglia. In contrast, the addition of CX3CL1 exacerbated hypoxia-induced endocytosis in WT microglia. These results suggest that the CX3CL1/CX3CR1 signal upregulates the hypoxia-induced microglial phagocytosis of synapses by promoting microglial endocytosis.

## Discussion

**FIGURE 7:**
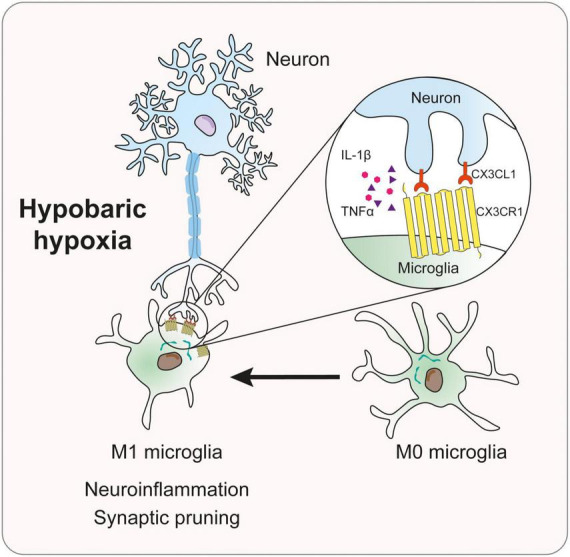
Mechanism diagram of CX3CL1/CX3CR1 signal in memory impairment of mice induced by hypobaric hypoxia.

The current study investigated the mechanism of the CX3CL1/CX3CR1 signal in memory impairment in mice induced by simulated high-altitude exposure. The major experimental findings were summarized as the following: (1) HH exposure induces microglia activation, and synaptic loss, and causes memory loss in mice; (2) HH induces M1-type polarized microglia and activates CX3CL1/CX3CR1 signal; (3) CX3CR1- deficiency significantly attenuates microglial activation, synaptic loss, and memory impairment induced by HH; (4) hypoxia-induced microglia polarization was dependent on CX3CR1, and CX3CL1 exacerbated hypoxia-induced M1-type microglia; and (5) hypoxia-induced the uptake of synapses through the upregulation of microglial phagocytosis. These findings support our hypothesis, suggesting that HH exposure activated CX3CR1/CX3CL1 signal, causing neuroinflammation and synaptic loss in the hippocampal region of mice by inducing M1-type microglia, which in turn triggered cognitive impairment (Figure 7).

Previous studies have reported that both acute and chronic HH cause cognitive impairment with complex mechanisms, including impaired brain self-regulation, glial vascular defects, oxidative stress, and neuroinflammation, which eventually translate into cognitive deficits (Wang M. et al., 2022). Zhou et al. (2021) subjected 8-week-old C57BL/6 mice to simulated 6000 m altitude exposure for 14 days and observed cognitive function impairment, PSD95 downregulation, and dendritic density decline. Xu et al. (2014) demonstrated the notable impairment of cognitive function in rats subjected to simulated altitude exposure of 3600 m. Experimental data from Ji et al. (2021b) indicated that after 4 and 8 weeks of 4300 m altitude exposure, the hippocampus and cortical neurons of rats showed swelling of neuronal mitochondria and an increase in neuronal apoptosis. This resulted in excitotoxicity, which eventually led to neurological damage and cognitive impairment (Ji et al., 2021b). After an 8-week exposure to 4300 m altitude, increased neuronal apoptosis and abnormal expression of cysteine-3 protein were observed, which led to the cognitive impairment of rats (Ji et al., 2021a). Zhao et al. (2019) gave rats 6000 m-altitude exposure for 24 h and observed neuronal damage and apoptosis in their hippocampal CA1 neuron. In addition, the mice developed memory impairment with no significant effect on anxiety. These results were consistent with our findings from the MWZ experiments that indicated both acute and chronic plateau hypoxia exposure cause memory impairment in animals, but exert no significant effect on anxiety. However, the pathological mechanisms underlying the altered cognitive function due to HH exposure are not fully understood to date. In the current study, we further concluded that HH reduced neuronal synapses associated with the M1-type polarization of microglia. In a previous study, we reported that acute HH exposure induces microglia activation and enhances phagocytosis (Wang X. et al., 2022). However, the findings of our present study confirmed that HH exposure induced M1-type microglia and upregulated phagocytosis dependent on CX3CL1/CX3CR1 signal. Thus, valuable insights into new mechanisms of neuroinflammation and neural injury triggered by HH exposure are obtained.

CX3CL1/CX3CR1 is an important pathway involved in the crosstalk between neurons and microglia, which functions differentially in acute and chronic brain injuries. Several studies have demonstrated that CX3CR1 activation is detrimental in cases of acute brain injury. In cerebral ischemia and spinal cord injury models, CX3CR1 deficiency exhibited neuroprotection by improving neural recovery and reducing neuroinflammation (Dénes et al., 2008; Cipriani et al., 2011; Donnelly et al., 2011; Fumagalli et al., 2013; Liu et al., 2015). Du et al. (2020) also found that the CX3CL1/CX3CR1 downregulation in a model of cerebral ischemia substantially promoted oligodendrocyte progenitor cell maturation and myelin regeneration by inhibiting microglia activation and reversed cognitive impairment. Febinger et al. (2015) reported that CX3CR1 plays a protective role in pre-acute brain injury, while it assumes a damaging role in late acute injury. However, in chronic neurological injury, the depletion of CX3CL1/CX3CR1 may contribute to neurological damage. Gabandé-Rodríguez et al. (2020) reported that CX3CR1 knockout mice demonstrated a significant increase in the number of microglia in the DG region and a decrease in dendritic spine density, while the synaptic pruning of microglia decreased during aging. Liu W. et al. (2020) found that CX3CR1 deficiency impedes neuronal communication with microglia. Rogers et al. (2011) demonstrated that CX3CR1 deficiency led to contextual fear conditioning and learning memory function. The group also indicated that the disruption of CX3CL1 leads to impaired cognitive function and synaptic plasticity through the upregulation of IL-1β expression (Rogers et al., 2011). These findings lead to the inference that the CX3CL1/CX3CR1 signal is very complex and the mechanism of action of CX3CL1/CX3CR1 signaling pathway in neurological disorders is bidirectional, which may be associated with disease progression and the alteration of multiple downstream pathways. Herein, we demonstrated that CX3CR1 deficiency notably attenuated the neuroinflammation and improved neurological recovery, suggesting that the cognitive impairment caused by the HH exposure was more biased toward acute brain injury. Considering the bidirectional role of the CX3CL1/CX3CR1 signal, we speculate that CX3CR1 may act differently in the mediation of cognitive dysfunction in the context of chronic hypoxic exposure, which warrants further study.

Our *in vivo* experiments revealed that simulated high-altitude exposure induced microglia to M1-type polarization. We further verified whether the M1-type microglia polarization was hypoxia-dependent. The changes in cell function caused by hypobaric hypoxia exposure are always directly caused by hypoxia. In the past studies, 1% O_2_ exposure welly constructed the *in vitro* model of altitude exposure (Wang et al., 2017, 2019; Wang M. et al., 2022; Jiang et al., 2021). Consistent with the *in vivo* results, the M1-type marker CD86 and pro-inflammatory factor IL-1β significantly increased post-hypoxic microglia. Interestingly, hypoxia did not affect the M2-type marker CD206 levels, which is different from the *in vivo* results. This suggests that the multicellular interactions and *in vivo* conditions confer a complex environment to the microglia, resulting in varying hypoxic responses. Butturini et al. (2019) showed that hypoxia induces M1-type polarization of microglia, triggering oxidative stress and pro-inflammatory cytokine release. Ha et al. (2021) reported that hypoxia at 1% O_2_ could increase the pro-inflammatory cytokine release by activating Toll-like receptor 4/NF-κB signal and upregulate NOD-like receptor thermal protein domain associated protein 3 expression, thereby activating pro-inflammatory microglia. In a rat model of cerebral under perfusion, Mao et al. (2020) found that post-transcriptional downregulation of miR-195 induced a CX3CL1/CX3CR1 upregulation accompanied by M1-type microglia. The aforementioned studies demonstrate that hypoxia or ischemia triggers M1-type microglia through multiple mechanisms. Our study further identified hypoxia-induced M1-type microglia to be dependent on CX3CR1, suggesting a critical regulatory role of CX3CR1 in hypoxic response, and providing a possible target for hypoxic encephalopathy treatment. In addition, we identified that CX3CL1 exacerbated hypoxia-induced M1-type microglia, suggesting the possibility of CX3CL1 having a role similar to hypoxia, both causing pro-inflammatory-type alterations in microglia through downstream activation CX3CR1. Several studies have reported that hypoxic pre-adaptation may have a protective effect on neuron function by moderately activating microglia. For example, Liu et al. demonstrated that hypoxic pre-adaptation induces M2-type microglia by inhibiting the Toll-like receptor 4/NF-κB signal and activating the PI3K/AKT signal (Liu W. et al., 2020), thus indicating a bidirectional effect of hypoxia. However, the regulatory role of the CX3CR1 signal during pre-adaptation has not been reported and this aspect deserves further investigation.

The results of our *in vivo* experiments implied that the reduced neurosynapses in the post-HH mouse brain may have a significant relationship to the upregulation of microglial phagocytosis. Microglia actively phagocytose and eliminate synaptic material during development and critically contribute to synaptic pruning during postnatal development in mice. Furthermore, defective microglia function leads to synaptic abnormalities in several neurodevelopmental disorders (Paolicelli et al., 2011). Our previous studies revealed that hypoxia causes the upregulation of microglia phagocytosis. While hypoxia enhances microglia phagocytosis by upregulating clathrin- and caveolin-dependent phagocytosis-related protein (Wang X. et al., 2022), it simultaneously enhances the microglial metabolism of phagosomes by enhancing caveolin-1-associated autophagy levels (Xue et al., 2022). We hypothesized that HH exposure could potentially lead to a reduction in synapses by enhancing the ability of microglia to phagocytose synapses. Cornell et al. reported that microglia regulate phagocytosis and synapse elimination through CX3CR1 and the CX3CR1/CX3CL1 signal regulates the microglial phagocytosis of oligodendrocyte progenitor cells during development (Cornell et al., 2022). Gunner et al. (2019) observed that CX3CR1 regulates microglia-mediated synaptic phagocytosis in the thalamocortex and the CX3CR1 knockout mice are severely deficient in synaptic phagocytosis and synaptic remodeling. These studies suggest a crucial regulatory role of CX3CR1/CX3CL1 in microglial phagocytosis of synapses. Our study demonstrated that hypoxia-induced increase in microglial phagocytosis of synapses was dependent on CX3CR1 and the exogenous addition of CX3CL1 exacerbated microglial phagocytosis of synapses under hypoxic conditions. Further investigation revealed that the activation of CX3CR1 increased microglial endocytosis, thus confirming a key regulatory role for CX3CR1 in microglial phagocytosis.

CX3CL1/CX3CR1 signal has been extensively researched on as an important mediator of inflammatory responses in neurological diseases. In a mouse model of Alzheimer’s disease, CX3CR1 deficiency was reported to trigger microglia dysfunction, and exacerbate neurotoxicity and phosphorylation of tau, leading to neuronal synaptic dysregulation and the impairment of memory function (Puntambekar et al., 2022). In an amyotrophic lateral sclerosis model, lack of CX3CL1/CX3CR1 signal in the CNS activates the NF-κB pathway, with increased microglia activation and neuronal damage (Liu et al., 2019). In contrast, in a cerebral ischemia model, CX3CL1/CX3CR1 downregulation significantly reversed cognitive deficits by inhibiting microglia activation and promoting oligodendrocyte progenitor cell maturation and myelin regeneration (Du et al., 2020). In a sleep deprivation model, CX3CL1/CX3CR1 deficiency attenuated inflammatory activity and synaptic pruning, which alleviated sleep deprivation-induced cognitive dysfunction (Xin et al., 2021). However, our findings confirmed that CX3CR1 deficiency had a protective effect on HH-induced memory impairment. Moreover, CX3CR1 was significantly upregulated and M1-type polarized in microglia, suggesting the therapeutic relevance of CX3CR1 in other diseases induced by plateau exposure. For example, in our previous study of high-altitude cerebral edema, plateau hypoxia caused increased microglia activation and exacerbated brain edema (Chen et al., 2022). Thus, CX3CR1 inhibition might be a potential therapeutic option for the disease.

This study identifies for the first time the role of CX3CR1 in high-altitude-induced cognitive impairment. Especially under acute plateau exposure, CX3CR1 inhibition may be an effective therapeutic approach, thus broadening the horizon for the treatment of cognitive impairment. Owing to the bidirectional role of CX3CR1 in acute and chronic brain injury, understanding the role of CX3CR1 in chronic plateau exposure requires further exploration. However, based on the complexity of the regulatory mechanism of cognitive impairment, it can be concluded that CX3CL1/CX3CR1 is only one of the factors affecting high-altitude-induced cognitive impairment. Several other factors and more related mechanisms need to be further elucidated.

## Data availability statement

The original contributions presented in this study are included in the article/Supplementary material, further inquiries can be directed to the corresponding authors.

## Ethics statement

This animal study was reviewed and approved by the Animal Care and Use Committee of Nantong University (Approval ID: S20220219-007).

## Author contributions

XW and LZ contributed to the conception and design of the study. YN, YX, and BW carried out experiments, organized the database, and performed the statistical analysis. XW wrote the first draft of the manuscript. YL and QL wrote sections of the manuscript. LZ completely revised the manuscript. All authors participated in reading the manuscript and approved the submitted version.
