# Supplementary material for: CX3CL1/CX3CR1 signal mediates M1-type microglia and accelerates high-altitude-induced forgetting
Source: Front Cell Neurosci. 2023 May 10;17:1189348. doi: 10.3389/fncel.2023.1189348 (PMC10206058; doi:10.3389/fncel.2023.1189348)
Supplement: Supplementary file 1 [file Data_Sheet_1.docx]

Supplementary Material

**CX3CL1/CX3CR1 signal mediates M1-type microglia and accelerates high-altitude-induced forgetting**

**Xueting Wang*, Yuqi Xie, Yun Niu, Baolan Wan, Yapeng Lu, Qianqian Luo, Li Zhu***

*** Correspondence:** Li Zhu, Ph.D. E-mail: zhulizhou@ntu.edu.cn;

Xueting Wang, Ph.D. E-mail: wangxueting@ntu.edu.cn

**Title for the Graphical Abstract:** Hypobaric-hypoxia promotes microglia polarization and synapse phagocytosis by activating CX3CL1/CX3CR1.

**1. All original data can be downloaded from the link: https://www.jianguoyun.com/p/DZwzQkMQvLqzCxiRuv0EIAA**

**Supplementary Figures and Tables**


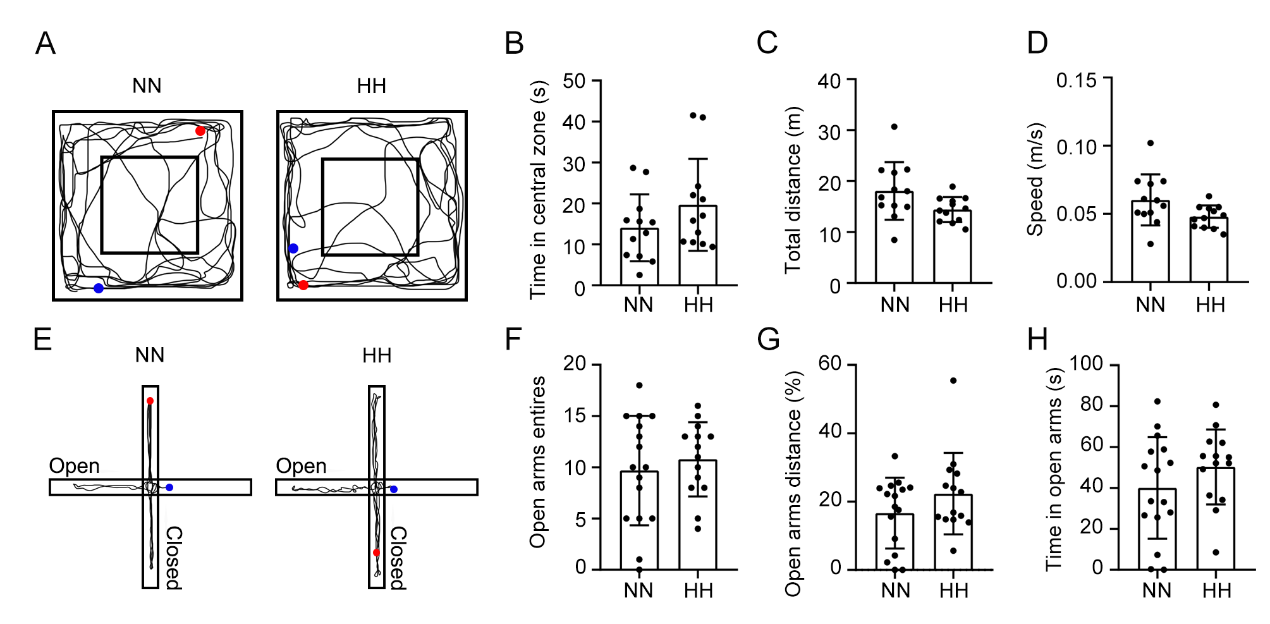


**Supp Figure 1 No effect of HH exposure on anxiety in mice. (A)** OFT trajectory of C57BL/6 mice after simulated exposure to 7000 m above sea level for 48 h. **(B)** Mice's residence time in the central region. **(C)** Total distance run by mice. **(D)** Mean speed of mice during OFT. **(E)** EPM trajectory of C57BL/6 mice after HH exposure for 48 h. **(F)** Number of times mice entered the open arms. **(G)** Ratio of the distance the mice moved in the open arms. **(H)** Residence time of mice in the open arms. n = 12.


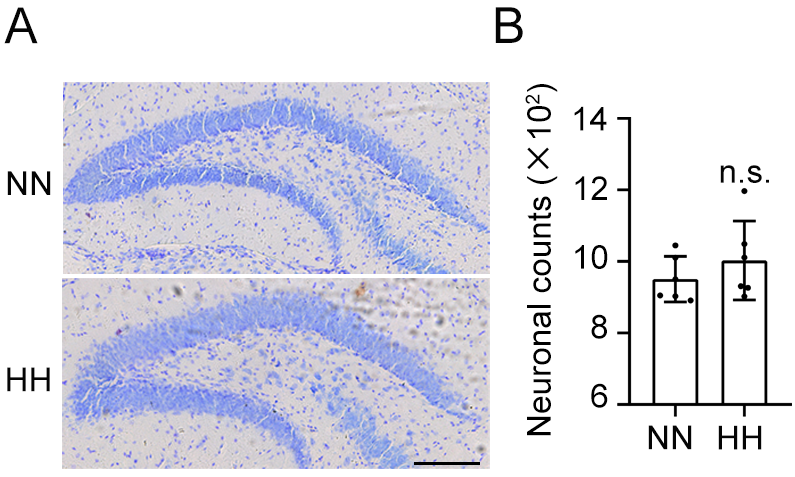


**Supp Figure 2 No effect of HH exposure on neurons number in hippocampal DG area. (A and B)** Nissl staining to label hippocampal DG area neurons and cell counting was performed by Image J software. Scale bar = 200 μm, n = 6. n.s. indicates no statistical difference.
